# Sharing good NEWS across the world: developing comparable scores across 12 countries for the neighborhood environment walkability scale (NEWS)

**DOI:** 10.1186/1471-2458-13-309

**Published:** 2013-04-08

**Authors:** Ester Cerin, Terry L Conway, Kelli L Cain, Jacqueline Kerr, Ilse De Bourdeaudhuij, Neville Owen, Rodrigo S Reis, Olga L Sarmiento, Erica A Hinckson, Deborah Salvo, Lars B Christiansen, Duncan J MacFarlane, Rachel Davey, Josef Mitáš, Ines Aguinaga-Ontoso, James F Sallis

**Affiliations:** 1 Institute of Human Performance, University of Hong Kong, Pokfulam Rd SAR, Hong Kong, 111-113, Hong Kong; 2 University of California, San Diego, 3900 Fifth Avenue, Suite 310, San Diego, CA, 92103, USA; 3 Ghent University, Watersportlaan 2, Ghent, 9000, Belgium; 4 Baker IDI Heart and Diabetes Institute, University of Queensland,, St Kilda Road, PO Box 6492, Central Victoria, 8008, Australia; 5 Catholic University of Parana, Rua Imaculada Conceição, Curitiba, 1155, Brazil; 6 Universidad de los Andes Carrera, 1ª N° 18A 10, Bogotá, Colombia; 7 Auckland University of Technology, Private Bag 92006, Auckland, 1142, New Zealand; 8 Emory University, 1518 Clifton Rd, CNR 7040E, Atlanta, GA, 30329, USA; 9 Institute of Sport Science and Clinical Biomechanics, University of Southern Denmark, Campusvej 55, Odense, M, 5230, Denmark; 10 Canberra University, University Drive Bruce, Canberra, ACT 2601, Australia; 11 Institute of Active Lifestyle’, Palacký University Tř. Míru, Olomouc, 115 77111, Czech Republic; 12 Public University of Navarra, Pio XII 36, Pamplona, Navarre, 31080, Spain; 13 Centre for Physical Activity and Nutrition Research (C-PAN), School of Exercise and Nutrition Sciences, Deakin University, 22 Burwood Highway, Burwood, VIC 3125, Australia

**Keywords:** Built environment, Questionnaire, Global, Confirmatory factor analysis, Pooled analyses

## Abstract

**Background:**

The IPEN (International Physical Activity and Environment Network) Adult project seeks to conduct pooled analyses of associations of perceived neighborhood environment, as measured by the Neighborhood Environment Walkability Scale (NEWS) and its abbreviated version (NEWS-A), with physical activity using data from 12 countries. As IPEN countries used adapted versions of the NEWS/NEWS-A, this paper aimed to develop scoring protocols that maximize cross-country comparability in responses. This information is also highly relevant to non-IPEN studies employing the NEWS/NEWS-A, which is one of the most popular measures of perceived environment globally.

**Methods:**

The following countries participated in the IPEN Adult study: Australia, Belgium, Brazil, Colombia, Czech Republic, Denmark, Hong Kong, Mexico, New Zealand, Spain, the United Kingdom, and the United States. Participants (N = 14,305) were recruited from neighborhoods varying in walkability and socio-economic status. Countries collected data on the perceived environment using a self- or interviewer-administered version of the NEWS/NEWS-A. Confirmatory Factor Analysis (CFA) was used to derive comparable country-specific measurement models of the NEWS/NEWS-A. The level of correspondence between standard and alternative versions of the NEWS/NEWS-A factor-analyzable subscales was determined by estimating the correlations and mean standardized difference (Cohen’s d) between them using data from countries that had included items from both standard and alternative versions of the subscales.

**Results:**

Final country-specific measurement models of the NEWS/NEWS-A provided acceptable levels of fit to the data and shared the same factorial structure with six latent factors and two single items. The correspondence between the standard and alternative versions of subscales of *Land use mix* – *access*, *Infrastructure and safety for walking*/*cycling*, and *Aesthetics* was high. The Brazilian version of the *Traffic safety* subscale was highly, while the Australian and Belgian versions were marginally, comparable to the standard version. Single-item versions of the *Street connectivity* subscale used in Australia and Belgium showed marginally acceptable correspondence to the standard version.

**Conclusions:**

We have proposed country-specific modifications to the original scoring protocol of the NEWS/NEWS-A that enhance inter-country comparability. These modifications have yielded sufficiently equivalent measurement models of the NEWS/NEWS-A. Some inter-country discrepancies remain. These need to be considered when interpreting findings from different countries.

## Background

Most studies of physical activity and built environments have been conducted in the USA, Australia and Western Europe, with recent studies extending findings to Japan [1], Colombia [2], China [3], Brazil [4], and elsewhere [5,6]. Though there have been important consistencies in the results [7], it is not possible to interpret different patterns of association by country because common methods were not employed. Further, the limited variability in environmental exposures and physical activity within countries may have underestimated the strength of association [7]. International evidence about the associations of the built environment with physical activity could inform international and national policies and guide the implementation of international health strategies, such as those from the World Health Organization [8]. Only international studies using comparable methods can establish the extent to which environment and policy associations with physical activity are generalizable across country or are country-specific. Such findings could inform evidence-based international and country-specific interventions to increase physical activity that could help underpin initiatives on the prevention of obesity and other non-communicable diseases that are high in developed countries and growing rapidly in developing countries [9,10].

One of the main aims of the IPEN (International Physical Activity and Environment Network) Adult project is to conduct multi-country pooled analyses using comparable measures to estimate associations of perceived attributes of the neighborhood environment with physical activity and health-related outcomes across 12 countries [11]. The Neighborhood Environment Walkability Scale (NEWS) [12] and its abbreviated form (NEWS-A) [13] were selected as measures of perceived neighborhood characteristics hypothesized to be related to physical activity, especially walking (e.g., land use mix, street connectivity, and traffic safety). The NEWS and NEWS-A have been adapted and/or translated for use in various countries [14-21]. Both the original and adapted/translated versions have shown acceptable test-retest reliability, concurrent validity with respect to objective environmental measures, and some evidence of criterion validity with respect to physical activity outcomes [12,15-18,21-24].

To date, four studies have established measurement models of factor analyzable items of the original [13,25] and adapted versions of the NEWS and/or NEWS-A [16,17] based on Confirmatory Factor Analyses (CFA). The CFA models describe the patterns of associations between items and their underlying latent constructs (e.g., street connectivity or aesthetics), thereby providing recommendations on how to summarize and score participants’ responses on the subscales [13,25]. Specifically, CFA can evaluate the extent to which responses to questionnaire items (e.g., perceived high crime rate, feeling unsafe to walk in the neighborhood during the day, or at night) that are hypothesized to measure the same construct, *aka* latent factor (e.g., safety from crime), share common variance. For each questionnaire item, CFA yields standardized factor loadings that indicate the magnitude and direction of associations between the responses on the items and their underlying latent construct. For example, a standardized factor loading of −0.85 for the item “perceived high crime rate” on the latent factor of crime safety would indicate that its responses are strongly negatively correlated with the factor.

All four studies that conducted a CFA of the NEWS/NEWS-A used a two-stage cluster sampling strategy to recruit participants from selected areas (i.e., selected study areas from which participants were recruited), they distinguished individual- from area-level measurement models - the former based on within-area differences and the latter on between-area differences in responses to the individual items. Because individual-level measurement models are more reflective of how perceptions of environmental characteristics group into factors and, thus, are likely more generalizable across populations than their area-level counterparts, it has been suggested that the NEWS and NEWS-A be scored according to the individual-level models [13,16,17,25]. The full and abbreviated versions of these instruments showed similar individual-level measurement models including the following multi-item latent factors: land use mix - access to services; street connectivity; infrastructure for walking/cycling; aesthetics; traffic safety; and safety from crime. Yet, several between-study discrepancies were noted, which might be attributed to differences in items or somewhat limited generalizability of specific measurement models to other geographical or cultural settings [25].

A prerequisite for conducting pooled analyses of multi-country data is the use of common protocols, including comparable exposure and outcome measures. In the case of the IPEN Adult project, a requirement for a country’s inclusion was measurement of perceived neighborhood attributes using the NEWS or NEWS-A, representing one of the main exposure measures. However, IPEN Adult was not a multi-center study that was funded at the outset with all countries required to follow an exact protocol. To optimize resources, some IPEN countries were able to receive local funding and proceed with their study before a funded coordinating center was in place to implement tight quality control. This funding model enabled more countries to contribute data, strengthened the study, and allowed countries some level of flexibility in matching the protocol to the local context, thereby making the study more relevant to their national situation. However, the downside was lack of comparability in some study elements, including the set of NEWS items used. Hence, the aim of the present paper was to compare subsets of comparable NEWS/NEWS-A items used across the 12 IPEN countries and, based on empirical evidence on their CFA-derived individual-level measurement models, propose scoring protocols that maximize cross-country comparability of responses. CFA-based NEWS/NEWS-A scores with demonstrated comparability across countries could then be used for either pooled analyses combining countries, or for study-specific non-pooled analyses, thereby facilitating both cross-study and cross-country comparisons. This information is not only important to studies included in the IPEN initiative, it is also highly relevant to other researchers who used or will use the NEWS/NEWS-A, which are currently the most popular measures of perceived neighborhood environment worldwide. Additionally, this paper also proposes a relatively simple analytical approach that can be used to create comparable measures for multi-country pooled analyses, when some deviations in the measurement protocol exist across study sites.

## Methods

### Neighborhood selection

The IPEN Adult study is an observational epidemiologic multi-country cross-sectional study. Twelve countries participated: Australia, Belgium, Brazil, Colombia, Czech Republic, Denmark, Hong Kong, Mexico, New Zealand, Spain, the United Kingdom, and the United States. Study participants were selected from neighborhoods chosen to maximize the variance in neighborhood walkability and Socio-Economic Status (SES); this occurred in all countries except Spain, where neighborhood SES was not available. The goal of the study design was to have equal numbers of neighborhoods stratified as follows: high walkable/high SES, high walkable/low SES, low walkable/high SES, and low walkable/low SES. For selection of study neighborhoods, all countries except Spain used a neighborhood walkability index that was objectively defined using Geographic Information Systems data at the smallest administrative unit available. A neighborhood walkability index for the whole area of study was first developed [26]. Then, neighborhoods with relatively lower and higher walkability index scores by lower and higher SES indicators were selected. In nine countries, participants were recruited across the seasons to control for variations in weather that may affect physical activity. In six countries, participants were recruited equally across the neighborhoods by season. The details for each country can be found elsewhere [11].

### Recruitment and participants

The required recruitment strategy was systematic selection of participants with addresses in the chosen neighborhoods. Adults living in the selected neighborhoods were contacted and invited to complete surveys on their physical activity and perceptions of the environment. Study dates ranged from 2002 to 2011. Each country obtained ethical approval from their local institutions and all participants provided informed consent. Age ranges for recruitment ranged from 15–84 years. Four countries recruited participants by phone and mail, and eight of the studies contacted households in person. Databases of resident addresses from commercial and government sources were used for the phone and mail recruitment. For the in-person recruitment, standard procedures for identifying households and participants within a household were employed [27]. In Hong Kong, intercept interviews were conducted in residential areas where individual addresses were not available, for example, in large apartment buildings with restricted access. Six countries used monetary incentives, and four countries provided non-monetary incentives including feedback on physical activity [28]. Six countries employed self-report methods (mail and online surveys) to collect survey data, four countries used interviews, and two countries used both self-report and interview methods. Further details for the participant recruitment techniques and response rates across countries can be found elsewhere [11].

There was a total N = 14,309 participants, ranging from 512 – 2,650 individuals from each country (see Table 1). The mean participants’ age was 42.3 (SD = 12.9) years. Overall, 57.1% were women, 38% had a high school degree, 43.9% a college degree, and 59.6% were married or living with a partner. Demographic descriptive statistics for each country are also shown in Table 1.

**Table 1:** Overall and country-specific sample characteristics

**Countries**	**All countries**	**Australia**	**Belgium**	**Brazil**	**Colombia**	**Czech Republic**	**Denmark**	**Hong Kong**	**Mexico**	**New Zealand**	**Spain**	**United Kingdom**	**United States of America**
**Cities**		Adelaide	Ghent	Curitiba	Bogotá	Olomouc Hradec Králové	Aarhus	Hong Kong	Cuerna-vaca	North Shore; Waitakere; Wellington; Christchurch	Pam-plona	Stoke on Trent	Baltimore, Maryland; Seattle, Washington
**Overall N**^**1**^	14309	2650	1166	699	1000	512	642	984	677	2033	904	843	2199
**Age:**													
Mean	42.3	44.5	42.7	41.1	41.1	37.0	38.9	42.8	42.1	40.6	38.7	43.0	45.1
SD	12.9	12.3	12.6	13.2	14.5	15.0	13.9	12.6	12.6	12.3	14.2	13.3	11.0
	%	%	%	%	%	%	%	%	%	%	%	%	%
**Sex:**													
Male	42.9	36.0	47.9	46.9	36.3	38.3	43.3	39.1	44.6	42.0	44.8	43.9	51.8
Female	57.1	64.0	52.1	53.1	63.7	61.7	56.7	60.9	55.4	58.0	55.2	56.1	48.2
**Education:**													
Did not complete high school	18.1	23.8	4.4	28.8	36.5	21.3	7.5	40.3	43.5	5.0	7.5	34.0	1.6
High school graduate or some college	38.0	30.0	34.6	32.5	41.7	49.0	44.5	22.9	28.8	56.9	34.7	52.0	33.4
College graduate or more	43.9	46.3	61.0	38.8	21.8	29.7	48.0	36.8	27.6	38.1	57.8	14.1	65.0
**Marital Status:**													
Not married or living with a partner	40.4	43.5	26.6	42.1	46.9	45.9	34.6	41.5	35.3	35.8	47.0	55.2	37.9
Married or living with a partner	59.6	56.5	73.4	57.9	53.1	54.1	65.4	58.5	64.7	64.2	53.0	44.8	62.1

N^1^ for some variables is reduced due to missing data.

### Measures

#### Versions of the neighborhood environment walkability scale

##### General overview

The full and abbreviated versions of the NEWS and NEWS-A comprise 67 and 54 items, respectively [12,13]. They gauge the following perceived neighborhood attributes: (1) residential density; (2) land use mix – diversity; (3) land use mix – access; (4) street connectivity; (5) infrastructure and safety for walking; (6) aesthetics; (7) traffic safety; (8) safety from crime; (9) streets not having many cul-de-sacs; (10) physical barriers to walking; (11) parking difficult in local shopping areas; and (12) hilly streets in the neighborhood. The United States employed the original full NEWS; New Zealand used the NEWS-A; while the remaining 10 countries used various combinations of NEWS/NEWS-A items, in their original or slightly modified forms. All countries included at least some items gauging the first 10 neighborhood attributes listed above. All non-English versions of the instrument were forward-translated from English into the local language, culturally adapted (when needed), and back-translated into English. At least two expert raters reviewed all versions of the NEWS/NEWS-A and evaluated item content equivalence.

#### Subscales (original and adapted)

The *Residential density* subscale of the original NEWS and NEWS-A consists of six items rated on a 5-point scale (1 = none; 2 = a few; 3 = some; 4 = most; 5 = all) (Table 2). Eleven out of 12 countries used the original response scale, while Belgium used a 3-point scale (1 = none; 2 = some; 3 = many). For the purpose of this study, responses on this subscale were recoded to range from 0 to 4 (0 to 3 for Belgium; i.e., 0 = none; 2 = some; 3 = most). This was done to enhance the accuracy of the measure so that perceived absence of a specific type of density-related attribute (e.g., apartments or condos with 4–6 stories) would not positively contribute to the total residential density score. Six countries used all six original items. The remaining countries reduced the number of items by merging the content of adjacent items or by omitting those gauging the highest levels of residential density. Finally, Hong Kong added an item to account for extreme levels of residential density (high-rise buildings with more than 20 stories) (Table 2). Ratings on the original six items of this subscale are weighted relative to the average residential densities that they represent, these being 1, 12, 10, 25, 50, and 75, respectively [12]. Table 2 describes modifications to the scoring procedures of the original version of this subscale that will be adopted to make it comparable across IPEN countries (see column Proposed solutions). A summary residential density score is obtained by summing up all weighted items’ scores.

**Table 2:** Differences in NEWS/NEWS-A items* across 12 countries and proposed solutions to maximize comparability across countries

**Original NEWS/NEWS-A subscale and items**	**Differences / issues**	**Proposed solution**
**Residential density**	Original scale ranging from 1 to 5, with 1 denoting ‘none’. This yields positive density scores even in absence of residential buildings and sometimes similar density scores for environments with different densities.	Recode original response scale ranging from 1 to 5 to 0 = none; 1 = a few; 2 = some; 3 = most; 4 = all
	Belgium used a 3-point (none; some; many) rather than 5-point scale (none; a few; some; most; all) on all subscale items.	The Belgian response “some” will be given a value of 2 (as in the recoded 5-point scale), while “many” will be assigned a value of 3, corresponding to “most” on the recoded 5-point scale.
1. Detached single-family residences	None	NA
2. Townhouses or rows of 1–3 stories houses	Hong Kong combined this item with item 3.	This item and item 3 have similar density weights (12 and 10). The Hong Kong item will be given a mid-weight of 11. For all other sites, with the exception of the UK which does not have item 3, the item with the highest rating (between item 2 and 3) will be chosen to be included in the summary residential density score and given a weight of 11. If items 2 and 3 have equal ratings, one of them will be included in the calculations and given a weight of 11.
		The UK will have this item weighted by 11.
3. Apartments or condos with 1–3 stories	Hong Kong combined this item with item 2.	See comments for item 2.
	The UK combined this item with item 4.	For the UK site, see comment for item 4 below.
4. Apartments of condos with 4–6 stories	The UK combined this item with item 3.	Items 3 and 4 have very different weights (12 and 25, respectively). We will weigh this UK item by the mid-point of the two original weights (18.5), with the assumption that the two types of apartments have similar prevalence.
5. Apartments or condos with 7–12 stories	None	NA
6. Apartments or condos with >12 stories	Australia, Belgium, Denmark, the Czech Republic, and the UK did not include it because high-rise residential buildings were judged to have very low prevalence. Hong Kong modified this item to read “Apartments or condos with 12–20 stories”.	No action is required as such buildings have low prevalence in the study sites that omitted this item. The mean score on this item would have been close to 0 and, thus, not adding to the overall density score. Hong Kong modified this item to distinguish residential buildings with 12–20 stories from those with 20–50 stories, which are common in Hong Kong but not in other study sites.
7. Apartments or condos with >20 stories	Hong Kong added this item to the subscale.	This item will be given a weight of 100.
**Land use mix – access**		
1. Stores within easy walking distance	None	NA
2. Many places within walking distance	Belgium and the UK did not include this item.	For these two countries, the CFA and summary score on this subscale will be based on 2 rather than 3 items. Using data from other countries, ascertain the correspondence between scores based on the 2- and 3-item subscales.
3. Easy to walk to a transit stop	None	NA
**Street connectivity**		
1. Short distance between intersections	Belgium did not include this item but had an additional item “four-way intersections” that is part of the NEWS street connectivity subscale	Include the additional connectivity item in the Belgian CFA model and, using data from countries that had all three street connectivity items, ascertain the comparability of the two 2-item street connectivity scores and a connectivity score based on an item common to Belgium and other countries (item 2).
2. Many alternative routes	Australia did not include this item but had an additional item “four-way intersections” that is part of the NEWS street connectivity subscale	Include the additional connectivity item in the Australian CFA model and, using data from countries that had all three street connectivity items, ascertain the comparability of the two 2-item street connectivity scores and a connectivity score based on an item common to Australia and other countries (item 1)
**Infrastructure and safety for walking and cycling**		
1. Sidewalks	None	NA
2. Cars separating sidewalks and traffic	Hong Kong did not include this item. Belgium combined it with item 3.	For these countries, the CFAs and summary score on this subscale will be based on a smaller number of items. Using data from other countries, ascertain the correspondence between scores on the subscale based on different combinations of items.
3. Grass/dirt separating sidewalks and traffic	Hong Kong did not include this item. Belgium combined it with item 3.	As above (see item 2).
4. Street lights	None	NA
5. Walkers and bikers easily seen	Australia, Brazil, and Hong Kong did not have this item.	As above (see item 2)
6. Crosswalks and pedestrian signals	None	NA
**Aesthetics**		
1. Trees	Australia included a slightly different item from the NEWS: “trees give shade”.	Include the additional item in the Australian CFA model and, using data from countries that had this additional item, ascertain the comparability of the Australian 4-item version of the subscale with the original 4-item version.
2. Many interesting things to look at	Belgium and the UK did not include this item.	For these two countries, the CFA and summary score on this subscale will be based on 3 rather than 4 items. Using data from other sites, ascertain the correspondence between scores based on the 3- and 4-item subscales.
3. Many attractive natural sights	None	NA
4. Attractive buildings/homes	None	NA
**Traffic safety/hazards**		
1. Heavy traffic along nearby streets	Brazil and the Czech Republic included a slightly different item from the NEWS: “heavy traffic along the street”	Use the slightly different item as a substitute for item 1. Using data from other countries that included all relevant items, ascertain the correspondence between scores based on the common subscale and these two countries’ version of the subscale.
2. Slow traffic speed on nearby streets	Australia, Belgium, and the Czech republic included a slightly different item from the NEWS: “slow traffic speed on the street”	Use the slightly different item as a substitute for item 2. Using data from other sites that included all relevant items, ascertain the correspondence between scores based on the common subscale and these three sites’ version of the subscale.
3. Speeding drivers	Australia did not include this item.	The Australian CFA and summary score on this subscale will be based on 2 rather than 3 items. Using data from other countries, ascertain the correspondence between scores based on the 2- and 3-item subscales.
**Safety from crime**		
1. High crime rate	None	NA
2. Unsafe to walk during the day	None	NA
3. Unsafe to walk at night	None	NA
**Few cul-de-sacs**	None	NA
**Physical barriers to walking**	Some differences in examples of type of barriers to walking.	NA

*NEWS*, Neighborhood environment walkability scale; *NEWS-A*, *NEWS*, Abbreviated form; *NA*, Not applicable; *CFA*, Confirmatory factor analysis; *UK*, United Kingdom; *Except for the items of the land use mix – diversity scale, which as explained in the text, was modified to provide a good match for all sites.

The *Land use mix* – *diversity* subscale of the original NEWS and NEWS-A is assessed by the perceived walking proximity from home to 23 different types of destinations, with responses ranging from 1–5 minute walking distance (coded as 5, indicative of high walkability) to >30-min walking distance (coded as 1, indicative of low walkability). Given that the lists of destinations varied substantially across countries due to cultural and geographical idiosyncrasies, individual destinations were collapsed into nine destination categories common to all countries and 13 destination categories common to 11 out of 12 countries (the UK had nine of these categories) . The nine categories common to all countries were: supermarket, small grocery or similar stores, post office, any school, transit stop, any restaurant, park, gym or fitness facility, and other stores and services. The 13 categories common to 11 out of 12 countries also included: library, video store, drug store/pharmacy and bookstore. Summary scores of land use mix – diversity are obtained by averaging rating across the nine or 13 categories of destinations to give a 9-destination and a 13-destination average score, respectively.

The remaining seven perceived environmental attributes assessed by all IPEN Adult study sites were rated using the original 4-point Likert scale (1 = strongly disagree; 4 = strongly agree). Summary scores for these subscales are computed by averaging the scores on the corresponding items (reverse scored, when necessary in the direction consistent with higher walkability and safety). All countries included the single-item subscales of *Streets not having many cul*-*de*-*sacs* and *Physical barriers to walking* (e.g., canyons, railways, freeways), and the 3-item *Safety from crime* subscale of the NEWS-A. All countries, with the exception of Belgium and the UK, used all items of the *Land use mix* – *access* subscale of the NEWS-A (Table 2). The 2-item *Street connectivity* subscale of the NEWS-A was included in all studies except for Australia and Belgium, these having one common and an additional item. Nine countries included the complete *Aesthetics* and eight of them completed the full *Infrastructure and safety for walking*/*cycling* subscales of the NEWS-A. The full NEWS-A *Traffic safety* subscale was included in eight countries (Table 2). A few countries included one or two *Aesthetics* or *Traffic safety* items that, albeit not identical, were potentially suitable substitutes for the original NEWS-A items (Table 2).

#### Socio-demographic characteristics

For the purpose of this paper, the following self-reported socio-demographic characteristics were considered: gender, age, educational attainment, and marital status.

### Data analyses

#### Site-specific measurement models of the NEWS/NEWS-A

Individual-level, site-specific measurement models of the NEWS/NEWS-A were derived by conducting separate CFAs for each country on the responses to factor-analyzable items (all items except for those measuring Residential density and Land use mix – diversity). Area-level clustering effects arising from the two-stage sampling procedures used in all studies were accounted for by conducting CFAs on within-area variance/covariance matrices quantifying estimates of individual-level relationships between the items [17]. CFAs were based on the Maximum Likelihood Estimation method. *A priori* individual-level site-specific measurement models of the NEWS-A were formulated taking into consideration the available items across countries, their comparability (see Table 2), and findings of previous CFAs of the NEWS and NEWS-A [13,16,17,25]. Measurement models including the following factors and single items were estimated:

1. *Land use mix* – *access*: two common (to all countries) items for the Belgian and UK models; three common items for the remaining 10 countries

2. *Street connectivity*: two common items, Australia and Belgium having a different combination of items than the remaining countries

3. *Infrastructure and safety for walking and cycling*: six common items for eight countries; five common items for Australia and Brazil; another combination of five common items for Belgium; and three common items for Hong Kong

4. *Aesthetics*: four common items for nine countries; three common items for Belgium and the UK; three common and an alternative item for Australia (here, ‘alternative’ means not included in all countries)

5. *Traffic safety*: three common items for eight countries; two common and an alternative item for Brazil; one common and two alternative items for Australia; and another combination of one common and two alternative items for Belgium and the Czech Republic

6. *Safety from crime*: three common items for all countries

7. *Not many cul*-*de*-*sacs*: a single common item for all countries

8. *Physical barriers to walking*: a single common item for all countries.

These eight factors were assumed to be inter-correlated. Jöreskog and Sörbom’s [29] iterative model-generating approach was used to re-specify the models and was guided by an inspection of standardized factor loadings, standardized residual covariances, univariate Langrage multiplier tests, Wald tests, multivariate outliers, and theoretical considerations [29]. The goodness-of-fit of the measurement models was assessed using a combination of model-fit indices recommended by Hu and Bentler [30] and Kline [31], including the Comparative Fit Index (CFI), the Root Mean Square Error of Approximation (RMSEA), and the Standardized Root Mean Squared residual (SRMS). According to Hu and Bentler [30], values supportive of good model fit are ≥0.95 for CFI, ≤0.06 for RMSEA, and ≤0.08 for SRMR. Given that the CFI is sensitive to the magnitude of correlations between variables [31] and co-occurring environmental attributes sometimes show only modest associations [17,32], we treated CFI values ≥0.90 as indicative of acceptable levels of model fit if the other two fit indices met Hu and Bentler’s [30] stricter criteria. We also reported the χ^2^ test. EQS 6.2 [33] was used to conduct CFAs.

#### Comparability of various versions of NEWS-A subscales

The level of overlap between corresponding standard and alternative versions of the NEWS/NEWS-A factor-analyzable subscales was determined by assessing the strength of associations (Pearson correlation coefficient) and mean effect size difference (in the form of Cohen’s d) between them. This could be done using data from countries that had included items from both standard and alternative versions of the subscales. A correlation coefficient ≥0.80 (indicative of collinearity) [34] and an absolute Cohen’s d <0.25 (indicative of very small effect sizes) [35] were considered supportive of significant conceptual overlap between the versions of the subscales.

## Results

### Country-specific measurement models of the NEWS/NEWS-A

After deletion of up to six multivariate outliers per country, the *a priori* individual-level measurement models of the NEWS/NEWS-A showed acceptable fit to the data of seven out of 12 countries (Table 3). The CFI values for the remaining countries did not meet the set criterion (≥.90). An inspection of the standardized factor loadings, standardized residuals, and Wald tests revealed that in the case of the Czech Republic, Mexico, Spain, Denmark, and the United Kingdom, two items (Cars separating sidewalks and traffic; Grass/dirt separating sidewalks and traffic) did not significantly load, or loaded in the opposite direction than expected, on the latent factor they were supposed to measure (Infrastructure and safety for walking/cycling). These two items also showed lower than desirable standard loadings (<|.30|) [36] for data from Australia, Colombia, and New Zealand. It was, thus, decided to omit them from all measurement models in order to ensure cross-country comparability. Apart from excluding the two problematic items, all models were re-specified by allowing item error terms to be correlated (where appropriate) and constraining inter-factor correlations to zero where the data did not provide sufficient support for an association. All re-specified models fitted the data sufficiently well, with five measurement models also fully satisfying Hu and Bentler’s [30] stricter goodness-of-fit criteria (Table 3). All standard factor loadings were significant at a probability level of < .001 in the expected direction (Table 4), with nearly all of them exceeding an absolute value of 0.30 indicating a substantial relationship [36]. All measurement models shared the same structure with six latent factors and two single items (Table 4). The average inter-factor correlations were low. However, across the various models, the Street connectivity latent factor was moderately or highly correlated with *Land use mix* – *access* and *Infrastructure and safety for walking*/*cycling* (Table 4).

**Table 3:** **Goodness-of-fit indices for** ***a priori*** **and final re-specified individual-level, country-specific measurement models of the NEWS/NEWS-A**

	**A priori models**				**Final models**			
**IPEN country**	**χ**^**2**^**(df)**	**CFI**	**RMSEA (95% CI)**	**SRMR**	**χ**^**2**^**(df)**	**CFI**	**RMSEA (95% CI)**	**SRMR**
Australia	593 (161)	0.934	0.041 (0.037, 0.044)	0.040	534 (139)	0.934	0.042 (0.039, 0.046)	0.044
Belgium	914 (202)	0.856	0.056 (0.052, 0.059)	0.058	462 (141)	0.907	0.045 (0.040, 0.049)	0.055
Brazil	340 (181)	0.906	0.046 (0.040, 0.056)	0.055	249 (155)	0.927	0.040 (0.031, 0.049)	0.053
Colombia	351 (202)	0.928	0.044 (0.036, 0.051)	0.068	262 (175)	0.956	0.036 (0.026, 0.044)	0.065
Czech Republic	453 (201)	0.897	0.053 (0.046, 0.059)	0.066	357 (171)	0.915	0.049 (0.042, 0.056)	0.060
Denmark	491 (202)	0.901	0.048 (0.043, 0.053)	0.052	316 (179)	0.948	0.035 (0.029, 0.041)	0.047
Hong Kong	247 (142)	0.952	0.041 (0.032, 0.049)	0.044	248 (144)	0.953	0.040 (0.032, 0.048)	0.045
Mexico	630 (202)	0.862	0.056 (0.050, 0.062)	0.068	369 (177)	0.915	0.046 (0.039, 0.053)	0.065
New Zealand	722 (202)	0.896	0.043 (0.040, 0.047)	0.045	501 (173)	0.930	0.037 (0.033, 0.041)	0.042
Spain	716 (202)	0.876	0.057 (0.052, 0.061)	0.065	512 (174)	0.911	0.050 (0.045, 0.055)	0.060
United Kingdom	322 (161)	0.933	0.036 (0.030, 0.042)	0.044	234 (137)	0.956	0.031 (0.024, 0.037)	0.045
United States of America	581 (202)	0.940	0.042 (0.039, 0.046)	0.046	480 (173)	0.951	0.041 (0.036, 0.045)	0.046

All χ^2^ significant at the < .001 level; NEWS = Neighborhood Environment Walkability Scale; NEWS-A: NEWS – Abbreviated form; df = degrees of freedom; CFI = comparative fit index; *RMSEA*, Root mean square error of approximation; *SRMR*, Standardized root mean squared residuals; 95% CI = 95% confidence intervals.

**Table 4:** Re-specified individual-level country-specific measurement models (standardized factor loadings) of the NEWS/NEWS-A

	**IPEN country**											
	**AUS**	**BEL**	**BRZ**	**COL**	**CZR**	**DEN**	**HKG**	**MEX**	**NZL**	**SPA**	**UK**	**USA**
**Factors and items**	**Standardized factor loadings**											
***Land use mix – access (LA)***												
LA1. Stores within easy walking distance	0.76	0.34	0.67	0.53	0.54	0.67	0.73	0.87	0.55	0.66	0.71	0.75
LA2. Many places within walking distance	0.82	-	0.70	0.74	0.73	0.75	0.81	0.56	0.69	0.73	-	0.85
LA3. Easy to walk to a transit stop	0.39	0.52	0.34	0.52	0.57	0.36	0.60	0.74	0.39	0.32	0.63	0.42
***Street connectivity (SC)***												
SC1. Short distance between intersections	0.37	-	0.31	0.46	0.39	0.32	0.41	0.46	0.37	0.35	0.47	0.35
SC2. Four-way intersections in neighborhood*	0.37	0.54	-	-	-	-	-	-	-	-	-	-
SC3. Many alternative routes	-	0.30	0.34	0.53	0.65	0.64	0.58	0.29	0.64	0.65	0.43	0.52
***Infrastructure and safety for walking and cycling (IS)***												
IS1. Sidewalks	0.35	0.43	0.38	0.56	0.45	0.52	0.43	0.31	0.30	0.32	0.40	0.42
IS2. Street lights	0.53	0.32	0.52	0.33	0.47	0.49	0.52	0.63	0.44	0.64	0.55	0.59
IS3. Walkers and bikers easily seen	-	0.30	-	0.30	0.48	0.51	-	0.47	0.33	0.58	0.34	0.57
IS4. Crosswalks and pedestrian signals	0.34	0.32	0.45	0.31	0.43	0.45	0.39	0.29	0.33	0.51	0.29	0.32
***Aesthetics (AE)***												
AE1. Trees	-	0.37	0.37	0.31	0.36	0.39	0.48	0.35	0.33	0.35	0.30	0.38
AE2. Trees give shade*	0.44	-	-	-	-	-	-	-	-	-	-	-
AE3. Many interesting things to look at	0.71	-	0.68	0.67	0.81	0.76	0.42	0.58	0.71	0.66	-	0.74
AE4. Attractive natural sights	0.60	0.75	0.71	0.79	0.57	0.60	0.67	0.75	0.64	0.71	0.62	0.75
AE5. Attractive buildings / homes	0.61	0.51	0.54	0.44	0.59	0.60	0.58	0.54	0.62	0.68	0.73	0.71
***Traffic safety/hazards (TH)***												
TH1. Heavy traffic along the street*	-	0.91	0.40	-	0.58	-	-		-	-	-	-
TH2. Heavy traffic along nearby streets	0.67	-	-	0.36	-	0.53	0.30	0.37	0.43	0.62	0.46	0.70
TH3. Slow traffic speed on the street*	−0.30	−0.36	-	-	−0.45	-	-	-	-	-	-	-
TH4. Slow traffic speed on nearby streets	-	-	−0.56	−0.49	-	−0.43	−0.42	−0.37	−0.64	−0.34	−0.64	−0.61
TH5. Speeding drivers	-	0.31	0.55	0.56	0.48	0.52	0.81	0.68	0.70	0.45	0.66	0.44
***Safety from crime (CR)***												
CR1. High crime rate	0.65	0.71	0.49	0.78	0.74	0.67	0.63	0.77	0.62	0.74	0.65	0.71
CR2. Unsafe to walk during the day	0.60	0.69	0.55	0.64	0.61	0.67	0.70	0.62	0.55	0.69	0.58	0.54
CR3. Unsafe to walk at night	0.71	0.82	0.68	0.84	0.74	0.88	0.68	0.81	0.74	0.83	0.81	0.82
***Few cul-de-sacs (CS)***	SI	SI	SI	SI	SI	SI	SI	SI	SI	SI	SI	SI
***Physical barriers to walking (BW)***	SI	SI	SI	SI	SI	SI	SI	SI	SI	SI	SI	SI
**Average absolute inter-factor correlation**	0.15	0.11	0.25	0.21	0.18	0.17	0.17	0.13	0.19	0.17	0.18	0.22
**Maximum absolute inter-factor correlation: pairs of factors**	0.68	0.33	0.91	0.96	0.65	0.57	0.72	0.62	0.54	0.49	0.73	0.52
	IS-AE	IS-CR	LA-SC	SC-IS	SC-IS	SC-IS	SC-IS	LA-SC	LA-SC	LA-SC	LA-SC	TH-CR
**Pairs of inter-correlated error terms**	0	5	5	7	8	1	0	6	5	4	4	4

*NEWS*, Neighborhood environment walkability scale; *NEWS-A*, *NEWS*, Abbreviated form; *Alternative items as substitutes to ‘core’ NEWS-A items; *SI*, Single item; *AUS*, Australia; *BEL*, Belgium; *BRZ*, Brazil; *COL*, Colombia; *CZR*, Czech Republic; *DEN*, Denmark; *HKG*, Hong Kong; *MEX*, Mexico; *NZL*, New Zealand; *UK*, United Kingdom; *USA*, United States of America.

### Comparability of versions of the NEWS/NEWS-A subscale

Due to differences in items and, hence, measurement models (Table 4), the NEWS/NEWS-A subscales of certain countries departed from the standard versions (Table 5). Comparability analyses showed that there was a high level of correspondence between the standard and alternative versions of the following subscales: *Land use mix* – *access*, *Infrastructure and safety for walking*/*cycling*, and *Aesthetics*. Using data from countries that had data on all relevant items, strong associations and small differences in means were found between scores on these three standard and alternative subscales. The alternative version of the *Traffic safety* subscale used by Brazil was also highly comparable to the standard version. The level of comparability of the remaining two versions of the *Traffic safety* subscale (for Australia and for Belgium and Czech Republic) was marginally acceptable, with average correlations and/or Cohen’s d at the limits of the acceptable range of values. The multi-item alternative versions of the *Street connectivity* subscale for Australia and Belgium did not provide a good match to their standard counterpart. However, single-item versions showed higher, yet marginally acceptable, levels of correspondence (Table 5).

**Table 5:** Comparability of alternative versions of NEWS/NEWS-A subscales with the standard versions

**Subscale [version]**	**Items**	**Countries using specific version**	No. of countries used for comparisons*	**Average (min, max) correlation between standard and alternative versions**	**Average (min, max) effect size for difference between mean scores on standard and alternative versions**	**Comparable**
Land use mix – access [standard]	AL1, AL2, AL3	AUS, BRZ, COL, CZR, DEN, HKG, MEX, NZL, SPA, USA				
Land use mix – access [alternative]	AL1, AL3	BEL, UK	10	0.94 (0.91, 0.96)	−0.10 (−0.16, 0.02)	YES
Street connectivity [standard]	SC1, SC3	BRZ, COL, CZR, DEN, HKG, MEX, NZL, SPA, USA				
Street connectivity [alternative 1]	SC1, SC2	AUS	2	0.74 (0.65, 0.82)	0.24 (0.11, 0.36)	NO
Street connectivity [alternative 2]	SC2, SC3	BEL	2	0.68 (0.56, 0.79)	0.19 (0.01, 0.36)	NO
Street connectivity [alternative 3]	SC1	AUS	10	0.79 (0.66, 0.85)	0.10 (−0.13, 0.26)	MARGINALLY
Street connectivity [alternative 4]	SC3	BEL	10	0.79 (0.75, 0.85)	−0.11 (−0.26, 0.13)	MARGINALLY
Infrastructure and safety for walking/cycling [standard]	IS1, IS2, IS3, IS4	BEL, COL, CZR, DEN, MEX, NZL, SPA, UK, USA				
Infrastructure and safety for walking/cycling [alternative]	IS1, IS2, IS4	AUS, BRZ, HKG	9	0.94 (0.89, 0.96)	−0.08 (−0.19, 0.05)	YES
Aesthetics [standard]	AE1, AE3, AE4, AE5	BRZ, COL, CZR, DEN, HKG, MEX, NZL, SPA, USA				
Aesthetics [alternative 1]	AE1, AE4, AE5	BEL, UK	9	0.96 (0.95, 0.97)	−0.03 (−0.18, 0.09)	YES
Aesthetics [alternative 2]	AE2, AE3, AE4, AE5	AUS	2	0.97 (0.96, 0.97)	0.02 (−0.12, 0.16)	YES
Traffic safety/hazards [standard]	TH2, TH4, TH5	COL, DEN, HKG, MEX, NZL, SPA, UK, USA				
Traffic safety/hazards [alternative 1]	TH2, TH3	AUS	2	0.80 (0.79, 0.81)	−0.03 (−0.57, 0.51)	MARGINALLY
Traffic safety/hazards [alternative 2]	TH1, TH4, TH5	BRZ	2	0.94 (0.93, 0.94)	0.09 (0.06, 0.12)	YES
Traffic safety/hazards [alternative 3]	TH1, TH3, TH5	BEL, CZR	2	0.80 (0.75, 0.84)	0.24 (0.17, 0.30)	MARGINALLY

Effect size is represented by Cohen’s d (<|0.25| = very small effect size). *Countries that had available data on all items of the standard and alternative version(s) of the subscale. *NEWS*, Neighborhood environment walkability scale; *NEWS-A,* NEWS – Abbreviated form; *AUS*, Australia; *BEL*, Belgium; *BRZ*, Brazil; *COL*, Colombia; *CZR*, Czech Republic; *DEN*, Denmark; *HKG*, Hong Kong; *MEX*, Mexico; *NZL*, New Zealand; *UK*, United Kingdom; *USA*, United States of America.

## Discussion

The IPEN Adult project aims to conduct pooled analyses of associations of perceived environment with physical activity and health outcomes using data from 12 countries differing in built and social environments [11]. The significant between-country differences in socio-demographic, cultural, and environmental factors required cultural adaptations to the NEWS [12] or NEWS-A [13], the perceived neighborhood environment measures adopted by the IPEN. To assist pooled analyses, it was important to establish scoring protocols that maximize the amount of usable data and yield comparable NEWS/NEWS-A summary scores across countries. These protocols are also relevant to future studies using the NEWS/NEWS-A since they facilitate cross-study comparison and, hence, can contribute to a better understanding of environment-physical activity relationships.

Several between-country differences were observed on all subscales of the NEWS/NEWS-A with the exception of *Safety from crime* and two single-item subscales. As detailed earlier, differences in the *Residential Density* and *Land use mix* – *diversity* subscales were resolved by modifying the scoring protocols of their original versions so to maximize inter-country comparability. For the remaining factor-analyzable items, well-fitting, comparable, individual-level measurement models consisting of eight distinct constructs (Table 4) were derived after omitting two problematic items (Cars separating sidewalks and traffic; Grass/dirt separating sidewalks and traffic). These findings provide further support for the robustness and generalizability of the factorial structure of NEWS and its abbreviated form, NEWS-A.

Interestingly, the two poor-fitting items mentioned above had some of the lowest standard factor loadings in the measurement models of the original NEWS and NEWS-A [13,25] and the Australian version of the NEWS [16]. In the present analyses, one or both items did not substantially load on any latent factor of the measurement models for Australia, Colombia, Denmark, Mexico, Spain, and the UK. Additionally, for the Czech Republic and Mexico, the item “Cars separating sidewalks and traffic” showed an unexpected negative loading on the factor it was supposed to measure (Infrastructure and safety for walking/cycling). Indeed, the presence of parked cars along sidewalks may in some cases be indicative of higher volumes of vehicular traffic and, hence, reflective of traffic hazards rather than safety. This conjecture was confirmed by field observations by the two respective research teams. Given the ambiguous interpretation of these two items across IPEN countries, it was decided to exclude them from all country-specific measurement models. Thus, we do not recommend they be used in the IPEN pooled analyses, though they can still be relevant in within-country analyses.

An issue worth mentioning pertains to the strong correlations observed between *Street connectivity* and the factors of *Land use mix* – *access* and *Infrastructure and safety for walking*/*cycling*, exceeding 50% of shared variance in four countries. This raises multi-collinearity concerns for multi-predictor regression models including all three subscales as explanatory variables, at least with respect to the countries where this appears to be as significant problem (Brazil, Colombia, Hong Kong, and the UK). Yet, these correlations represent associations between latent factors rather than subscale scores, the latter being used in regression analyses. Latent factor scores include only items’ communalities (the items’ variances that are accounted for by a factor), while subscale scores representing the average rating on the relevant items also include items’ uniquenesses, which are in part random error variance [36]. Thus, correlations between latent factors are usually higher than those between the corresponding ‘raw’ subscale scores. Post-hoc analyses revealed that this was also the case in this study. Specifically, the correlations between the scores on the Street connectivity and the other two potentially collinear subscales ranged from 0.23 to 0.41. Hence, the simultaneous inclusion of these three subscales as explanatory variables in regression models should not create multi-collinearity problems.

As noted earlier, six out of 12 countries did not use standard versions of the NEWS/NEWS-A subscales. An analysis of the level of comparability of the various versions of the subscales revealed some potential concerns with the Australian and Belgian versions of (single-item) *Street connectivity* and *Traffic safety*, and the Czech version of *Traffic safety*. Specifically, the average correlations between the standard and alternative versions of the subscales only just met the adopted comparability criterion (≥0.80) and substantial variability was observed in the individual country-specific correlations. This has some implications for the interpretation of results from pooled analyses with respect to these subscales. Namely, eventual between-country (Australia, Belgium, and the Czech Republic vs. other countries) differences in associations of these two perceived environmental attributes with outcome measures (detectable in the form of significant country by perceived attribute interaction effects) may be in part due to measurement differences. Hence, caution will be needed in interpreting such significant interaction effects (if any), especially if they are small in magnitude.

Also of concern are the observed differences in mean scores between the standard and two alternative versions of the *Traffic safety* subscale. Although the alternative subscales had acceptable or marginally acceptable average effect sizes, some country-specific effect sizes were too large, suggesting the possibility that differences in scores between the standard and alternative subscales for Australia, Belgium, and the Czech Republic (if data on all relevant items had been available from these countries) might be substantial. Thus, pooled analyses of between-site differences in average perceived neighborhood environmental attributes will need to take into account the possibility that the presence or lack of differences in perceived traffic safety between these three sites and the remaining sites be due to differences in measures.

Given the above, we recommend that for the purpose of conducting pooled analyses on data from the 12 IPEN countries, the factor-analyzable NEWS/NEWS-A subscales be scored according to the respective country-specific measurement models presented in Table 4, with the exception of the Australian and Belgian versions of the *Street connectivity* subscale which, for these two countries, should consist of a single item (see algorithms presented in Table 6). The *Residential density* and *Land use mix* – *diversity* subscales should be scored according to the algorithms shown in Table 6, which are based on previously presented analyses and remarks (see Table 2 and Methods section). We also recommend that future studies employing country-specific versions of the NEWS/NEWS-A use the here-proposed protocols.

**Table 6:** Country-specific scoring of NEWS/NEWS-A subscales for pooled analyses

**Subscale [version]**	**Algorithm (item)**	**Country**
***Residential density***	If (RD2) > (RD3) then (RD1) + (RD2) * 11 + (RD4) * 25 + (RD5) * 50 + (RD6) * 75	BRZ, COL, MEX, NZL, SPA, USA
[standard]	If (RD3) > (RD2) then (RD1) + (RD3) * 11 + (RD4) * 25 + (RD5) * 50 + (RD6) * 75	
	If (RD2) = (RD3) then (RD1) + (RD2) * 11 + (RD4) * 25 + (RD5) * 50 + (RD6) * 75	
[alternative 1]	If (RD2) > (RD3) then (RD1) + (RD2) * 11 + (RD4) * 25 + (RD5) * 50	BEL (responses recoded; see Methods section), AUS, DEN, CZR
	If (RD3) > (RD2) then (RD1) + (RD3) * 11 + (RD4) * 25 + (RD5) * 50	
	If (RD2) = (RD3) then (RD1) + (RD2) * 11 + (RD4) * 25 + (RD5) * 50	
[alternative 2]	(RD1) + (RD2_HKG) * 11 + (RD4) * 25 + (RD5) * 50 + (RD6) * 75 + (RD7) * 100	HKG
[alternative 3]	(RD1) + (RD2) * 11 + (RD3_UK) * 18.5 + (RD5) * 50	UK
***Land use mix – diversity***	[(LD1) + … + (LD9)] / 9	All (Items reclassified; see Methods section)
[standard]		
[alternative 1]	[(LD1) + … + (LD13)] / 13	AUS, BEL, BRZ, COL, CZR, DEN, HKG, MEX, NZL, SPA, USA (Items reclassified; see Methods section)
***Land use mix – access***	[(LA1) + … + (LA3)] / 3	AUS, BRZ, COL, CZR, DEN, HKG, MEX, NZL, SPA, USA
[standard]		
[alternative 1]	[(LA1) + (LA3)] / 2	BEL, UK
***Street connectivity***	[(SC1) + (SC3)] / 2	BRZ, COL, CZR, DEN, HKG, MEX, NZL, SPA, UK, USA
[standard]		
[alternative 1]	(SC1)	AUS
[alternative 2]	(SC3)	BEL
***Infrastructure and safety for walking and cycling***	[(IS1) + … + (IS4)] / 4	BEL, COL, CZR, DEN, MEX, NZL, SPA, UK, USA
[standard]		
[alternative 1]	[(IS1) + (IS2) + (IS4)] / 3	AUS, BRZ, HKG
***Aesthetics***	[(AE1) + (AE3) + (AE4) + (AE5)] / 4	BRZ, COL, CZR, DEN, HKG, MEX, NZL, SPA, USA
[standard]		
[alternative 1]	[(AE1) + (AE4) + (AE5)] / 3	BEL, UK
[alternative 2]	[(AE2) + … + (AE5)] / 4	AUS
***Traffic safety/hazards***	[(TH2_R) + (TH4) + (TH5_R)] / 3	COL, DEN, HKG, MEX, NZL, SPA, UK, USA
[standard]		
[alternative 1]	[(TH2_R) + (TH3)] / 2	AUS
[alternative 2]	[(TH1_R) + (TH4) + (TH5_R)] / 3	BRZ
[alternative 3]	[(TH1_R) + (TH3) + (TH5_R)] / 3	BEL, CZR
***Safety from crime***	[(CR1_R) + (CR2_R) + (CR3_R)] / 3	All
[standard]		
***Few cul-de-sacs***	(CS)	All
[standard]		
***Physical barriers to walking***	(BW)	All
[standard]		

*NEWS*, Neighborhood environment walkability scale; *NEWS-A* ,NEWS – Abbreviated form; *AUS*, Australia; *BEL*, Belgium; *BRZ*, Brazil; *COL*, Colombia; *CZR*, Czech Republic; *DEN*, Denmark; *HKG*, Hong Kong; *MEX*, Mexico; *NZL*, New Zealand; *UK*, United Kingdom; *USA*, Unites States of America. _*R*, Reversed scored item; _*HKG*, Hong Kong-specific item; _*UK*, UK-specific item; the item codes correspond to those in Table 4.

### Limitations

The main limitations of this study pertain to differences in the participant recruitment procedures, survey administration mode, and the use of somewhat different versions of the NEWS/NEWS-A across countries. However, differences in the ways participants were recruited are unlikely to have had a systematic impact on the inter-item associations and, thus, measurement models of the NEWS/NEWS-A. Additionally, it is important to note that nearly all countries used the same stratified two-stage sampling procedure to recruit participants from neighborhoods selected on the bases of their socio-economic and walkability levels. This likely contributed to the recruitment of relatively comparable samples across countries in terms of educational attainment and exposure to low- vs. high-walkable environments, two characteristics that may impact the accuracy and variability of responses to measures of perceived neighborhood environment [15,37,38]. Self-administered surveys may have resulted in less socially desirable and more consistent data [39]. Yet, differences in survey findings across modes of administrations are generally small and more pronounced when examining sensitive topics [39]. However, the items included in the NEWS/NEWS-A (e.g., access to services and traffic safety) are unlikely to be perceived as delicate issues.

The fact that the 12 IPEN countries did not use exactly the same group of survey items precluded the conduct of a more rigorous assessment of the cross-country equivalence of the NEWS/NEWS-A, whereby measurement model fit would be assessed by progressively increasing the number of constrained parameters (i.e., parameters would be constrained to be equal across IPEN countries), starting from factor loadings and finishing with the variances of errors terms [40]. With the available data, we were only able to demonstrate cross-country configural equivalence of the NEWS/NEWS-A, i.e., that all measurement models consisted of the same latent factors. This is an essential requirement for the conduct of pooled analyses based on NEWS/NEWS-A data.

## Conclusions

To improve inter-country comparability , and allow pooled analyses of data, in investigations of associations of perceived neighborhood environment with physical activity and health outcomes, we have proposed modifications to the original scoring protocol of the NEWS/NEWS-A and have established country-specific, comparable measurement models to be employed in future analyses. A few potential inter-country discrepancies remain with respect to the measurement of street connectivity and traffic safety, which need to be considered in the interpretation of findings based on pooled analyses and comparison of findings from different countries. We recommend that future studies using the NEWS/NEWS-A implement the here proposed scoring protocol to facilitate cross-study comparability and interpretation of the findings. Importantly, the analytical approach presented in this paper could also be used by other multi-site projects with variations in the measurement protocol and requiring optimization of data inclusion and comparability for pooled analyses and cross-site comparisons.

## Abbreviations

CFA: Confirmatory factor analysis; NEWS: Neighborhood environment walkability scale; NEWS-A: Neighborhood environment walkability scale-abbreviated version; IPEN: International physical activity and environment network; SES: Socio-economic status; SD: Standard deviation; CFI: Comparative fit index; RMSEA: Root mean square error of approximation; SRMR: Standardized root mean squared residual.

## Competing interests

The authors declare that they have no competing interests.

## Authors’ contributions

EC conceptualized the study and modifications to the scoring protocols, devised and conducted the confirmatory factor and comparability analyses, drafted the Results and Discussion sections and part of the Introduction and Methods sections; and is one of the PIs of the Hong Kong study. TLC is a co-investigator on the US study and Coordinating Center grant, had primary oversight for data management and processing of data from countries at the San Diego Coordinating Center, constructed the datasets used for analyses, and assisted in drafting the manuscript. KLC is the project manager for the US study and Coordinating Center grant and coordinated cross-country data collection efforts and data processing at the San Diego Coordinating Center. JK is a co-founder of the IPEN group and a co-investigator on the Coordinating Center grant and assisted in drafting the manuscript. IDB is co-founder of the IPEN group and PI of the Belgian study. NO co-founded the IPEN group and is PI of the Australian study. RSR coordinated and is PI of the study Brazilian study. OLS coordinated and is PI of the study in Bogotá (Colombia). EAH is co-investigator of the New Zealand arm of the IPEN study. DS coordinated and is co-investigator of the Mexican arm of the IPEN study. LBC coordinated and is co-investigator of the Danish study. DJM is the project manager and one of the PIs for the Hong Kong study. RD coordinated and is PI of the UK study. JM is co-investigator of the study conducted in the Czech Republic. IAO coordinated and is co-investigator of the study Spanish study. JFS co-founded the IPEN group, is PI of the US study and Coordinating Center grant. All authors reviewed and edited the manuscript, and approved the final manuscript as submitted.

## Pre-publication history

The pre-publication history for this paper can be accessed here:

http://www.biomedcentral.com/1471-2458/13/309/prepub
